# Rebamipide promotes lacrimal duct epithelial cell survival via protecting barrier function

**DOI:** 10.1038/s41598-020-58314-x

**Published:** 2020-02-03

**Authors:** Hiroshi Tanaka, Tomomichi Nakayama, Michiko Tsukamoto, Akihide Watanabe, Takahiro Nakamura, Norihiko Yokoi, Chie Sotozono, Shigeru Kinoshita

**Affiliations:** 1 Department of Ophthalmology, Kyoto Prefectural University of Medicine, Kyoto, Japan; 2 Department of Frontier Medical Science and Technology for Ophthalmology, Kyoto Prefectural University of Medicine, Kyoto, Japan

**Keywords:** Cell biology, Diseases

## Abstract

Nasolacrimal duct obstruction (NLDO) is thought to be due to inflammation and fibrosis of lacrimal duct epithelial cells (LDECs). Here we investigated the effect of rebamipide, a drug that is used for the protection of the mucosa and the treatment of gastritis and gastroduodenal ulcers, on LDECs, both *in vitro* and *in vivo*. In this study, LDECs were cultured from rabbit lacrimal duct tissues, and the barrier function of LEDCs was examined *in vitro* via transepithelial electrical resistance (TER) measurement, with or without interleukin (IL)-6 and/or rebamipide. For the *in vivo* examination, benzalkonium chloride (BAC) was injected into the rabbit lacrimal ducts, followed by the application of rebamipide or a placebo vehicle alone. The results of the *in vitro* examination revealed a significant decrease in TER in the group treated with IL-6 alone compared with the placebo-vehicle group (*p* < 0.05) and the group treated with IL-6 and rebamipide (*p* < 0.01). The results of the *in vivo* examination revealed that the infiltration of neutrophils under the basement membrane and the disruption of tight junction proteins with BAC injection and rebamipide attenuates the disturbance of tissue construction. These results suggest that rebamipide protects LDECs via an anti-inflammatory effect and preserves the barrier function of those cells.

## Introduction

It has been theorized that inflammation and age-related fibrosis of the lacrimal-duct epithelium are involved in the pathology of idiopathic nasolacrimal duct obstruction (NLDO)^1^. The common symptoms of NLDO include epiphora, discharges and dacryocystitis, an infection or inflammation of the lacrimal sac. Lacrimal stent intubation or dacryocystorhinostomy improves the symptoms, but these treatments are accompanied by surgical stress.

It has recently been reported that various tear cytokines, such as interleukin (IL)-2, IL-6, and IL-10, are involved in the pathology of primary acquired nasolacrimal duct obstruction (PANDO)^2^, and that IL-6 is also involved in congenital NLDO (CNLDO)^3^. Thus, the suppression of the level of inflammatory cytokines in tears is considered to be a key factor in the development of new methods for the treatment of NLDO.

Rebamipide, an oral drug that is used for the protection of the mucosa and the treatment of gastritis and gastroduodenal ulcers, is currently marketed in Japan as an ophthalmic suspension (Mucosta^®^; Otsuka Pharmaceutical Co., Ltd, Tokyo, Japan) for the treatment of dry eye. We previously reported that rebamipide has an anti-inflammatory effect and enhances the barrier function of cultured human corneal epithelial cells (CECs)^4^. In a previous study by Mimura *et al*., it was reported that the postoperative administration of rebamipide increases the rate of surgical success in patients who undergo lacrimal stent intubation for the treatment of PANDO^5^. However, the exact mechanism for the anatomical success in that study was not revealed.

In this present study, we investigated the barrier function and protective effect of rebamipide on rabbit lacrimal duct epithelial cells (LDECs), both *in vitro* and *in vivo*.

## Methods

### Animals

The animals used in this study were male adult Japanese white rabbits (body-weight range: 3.5–3.99 kg), and all animals were treated in accordance with the ARVO Statement for the Use of Animals in Ophthalmic and Vision Research. All experimental procedures were approved by the Committee for Animal Research at Kyoto Prefectural University of Medicine, Kyoto, Japan.

### *In vitro* examination

For the *in vitro* examination, rabbits were first sacrificed with an intravenous injection of pentobarbital. Next, lacrimal tissue samples were obtained, and LDECs were collected from those samples via the use of Dispase II (FUJIFILM Wako Pure Chemical Corporation, Osaka, Japan). First, the collected LDECs were then cultured on 6-well dishes with keratinocyte serum free medium (Defined Keratinocyte-SFM; Thermo Fisher Scientific, Inc., Waltham, MA), BEGM^™^ Bronchial Epithelial Cell Growth Medium (Lonza Group Ltd., Basel, Switzerland), and 8% fetal bovine serum (Thermo Fisher Scientific). The cells were then subcultured on 24-well Transwell Permeable Supports (Corning Inc., Corning, NY) until cell confluence was achieved. The transepithelial electrical resistance (TER) in the cells was then measured post confluence in 3 groups: Group A (0-ng/ml IL-6, 0-mM rebamipide); Group B (0.5-ng/ml IL-6, 0-mM rebamipide); Group C (0.5-ng/ml IL-6, 2-mM rebamipide) (n = 4 each) at 0-, 24-, and 72-hours after stimulation.

Second, the LDECs were subcultured on 24-well dishes until cell confluence was achieved. The cells were stimulated with 1% benzalkonium chloride (BAC) with 2-mM rebamipide or the vehicle for 72-hours and fixed in 4% paraformaldehyde, and investigated with immunohistochemistry. As a control, the cells were incubated with medium (n = 2 each).

### *In vivo* examination

We next investigated whether BAC affected the nasolacrimal duct epithelium *in vivo*, and whether rebamipide might prevent the damage of epithelium with BAC treatment. For the *in vivo* examination, the rabbits were first placed under general anesthesia via the injection of a cocktail containing ketamine and xylazine. Next, an LDEC damage model was created by injecting 250 µl of 1% BAC into the rabbit’s lacrimal duct once daily for 3 consecutive days. After verification that the LEDC damage model was successfully created, 250 μl of 2% rebamipide ophthalmic solution (Mucosta^®^ ophthalmic suspension UD2%) or the vehicle of rebamipide was injected (n = 3 each) simultaneously with the injection of 1% BAC. As a control, phosphate buffered saline (PBS) was injected into the lacrimal duct of 3 rabbits.

Next, lacrimal duct tissue was obtained and embedded in optimal cutting temperature compound (Tissue-Tek^®^ O.C.T.; Sakura^®^ Finetek Japan Co., Ltd., Tokyo, Japan). The tissue was then cut into 7-um sections, fixed in 4% paraformaldehyde, and investigated with histological examination and immunohistochemistry (n = 3 each). The thickness of the LDECs was then measured via the results of the histological examinations by another investigator, and the measurements were compared between the 3 groups.

### Immunohistochemistry examination

Immunohistochemistry staining was performed with mouse antibodies to zonula occludens (ZO) protein ZO-1, claudin (CLDN)1, and CLDN7 (1:100; Invitrogen) overnight at 4 °C. Next, the sections were incubated with a secondary antibody (Alexa Fluor 488 goat anti-mouse IgG; 1:1000; Invitrogen) for 1 hour at room temperature, and then mounted with Vectashield^**®**^ Antifade Mounting Medium (Vector Laboratories, Burlingame, CA) containing 4′,6 diamidino-2-phenylindole.

### Analysis via scanning electron microscopy (SEM)

For SEM analysis, the obtained tissue samples that were first fixed in 0.1 mol phosphate buffered 2% glutaraldehyde, then subsequently post-fixed in 2% osmium tetroxide for 2 hours in an ice bath. The specimens were then dehydrated in a graded ethanol and dried by frozen t-butyl alcohol in a vacuum. The freeze-dried specimens then underwent SEM examination (JEM-7500F; JEOL Ltd., Tokyo, Japan) after being coated with an osmium plasma ion coater device.

### Statistical analysis

Statistical analysis of the data was performed and basic descriptive statistics were calculated on all of the gathered data, with the values reported as mean ± SD. Results were compared among three groups by Bonferroni correction. A p value of <0.05 was considered statistically significant.

## Results

### Effect of rebamipide on the barrier function of LDECs *in vitro*

**Figure 1:**
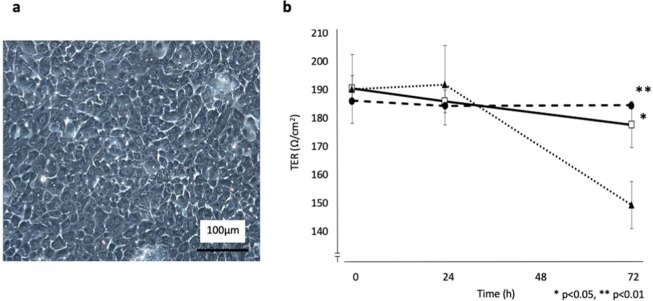
Cultivation of rabbit lacrimal duct epithelial cells (LDECs) and effects of rebamipide and inflammatory cytokine in the barrier function of these cells. (**a**) Phase contrast image of cultivated rabbit LDECs *in vitro*. Scale bar = 100 µm. (**b**) Rabbit LDECs were incubated with or without rebamipide; Group A (0-ng/ml IL-6, 0-mM rebamipide) (open square), Group B (0.5-ng/ml IL-6, 0-mM rebamipide) (closed triangle) and Group C (0.5-ng/ml IL-6, 2-mM rebamipide) (closed circle) for 0-, 24-, or 72-hours, after which transepithelial electrical resistance (TER) was measured (n = 4 each). Data are means ± SEM of representative of a total of four experiments (*p < 0.05, **p < 0.01) (Bonferroni correction).

We first investigated the effect of rebamipide on the barrier function of rabbit LDECs that were successfully cultured *in vitro* (Fig. 1a). The cells were incubated with or without 0.5-ng/ml IL-6 and 2-mM rebamipide, i.e., Group A: 0-ng/ml IL-6, 0-mM rebamipide, Group B: 0.5-ng/ml IL-6, 0-mM rebamipide, and Group C: 0.5-ng/ml IL-6, 2-mM rebamipide, and TER was then measured at 0-, 24-, and 72-hours post stimulation. At 0-, 24-, and 72-hours post stimulation, TER was 190.9 ± 21.5, 186.3 ± 6.1, and 178.1 ± 12.1 Ω/cm^2^, respectively, in Group A, 190.6 ± 12.1, 192.0 ± 14.7, and 149.9 ± 8.3 Ω/cm^2^, respectively, in Group B and 186.4 ± 4.3, 184.8 ± 4.0, and 184.8 ± 8.1 Ω/cm^2^, respectively, in Group C (Fig. 1b). At 0- and 24-hours post stimulation, no significant difference was observed among the 3 groups. At 72-hours post stimulation, in Group B, which was treated with IL-6, showed significant decrease in TER compared to Group A (*p* < 0.05) and Group C (*p* < 0.01).

### Effect of rebamipide on damaged LDECs *in vivo*

**Figure 2:**
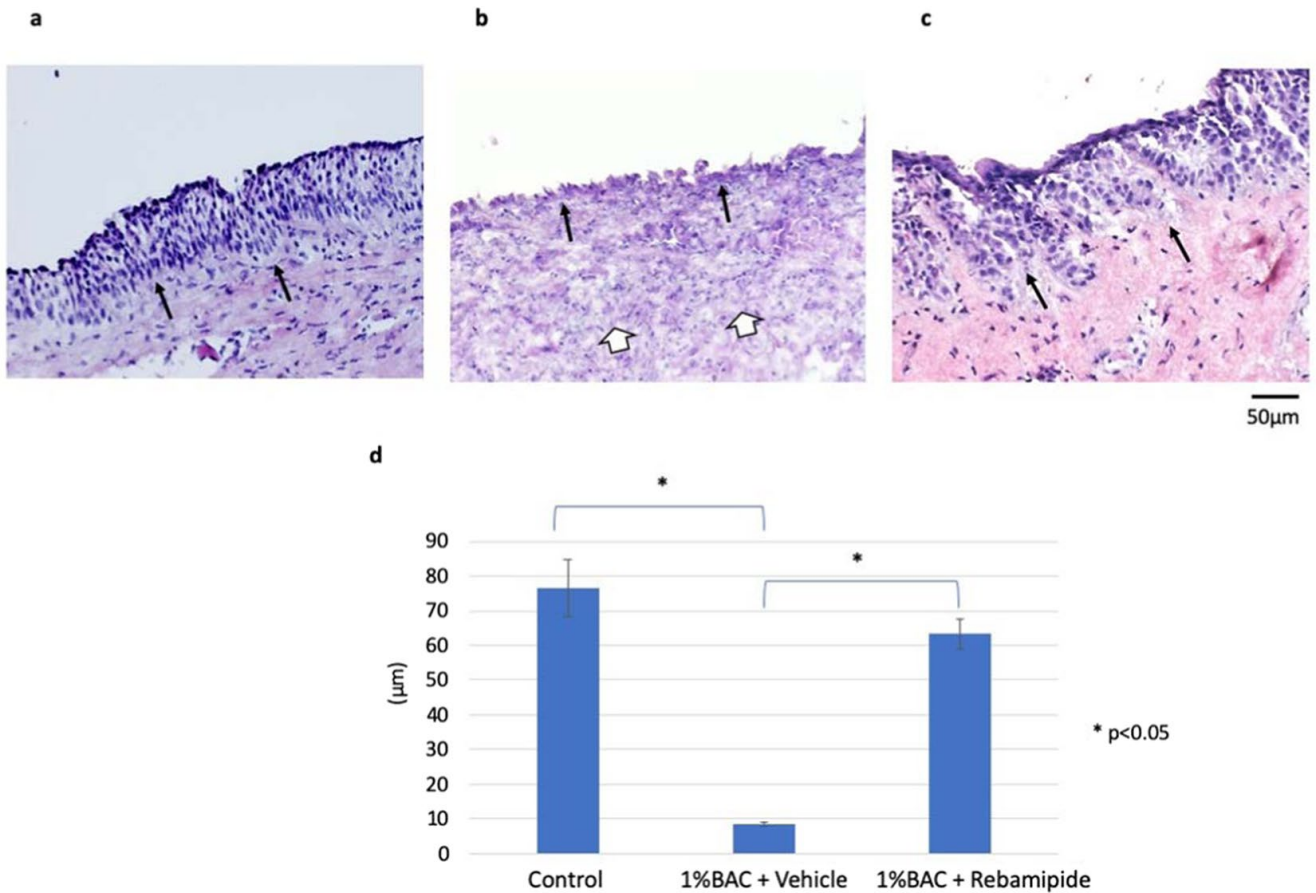
Histological images illustrating the thickness of the rabbit lacrimal duct (n = 3 each). Histological image of a rabbit lacrimal duct in the control group (**a**). LDECs in the 1% benzalkonium chloride (BAC) and the vehicle of rebamipide injected group were significantly disrupted and shortened (**b**) compared to the 1% BAC and rebamipide injected group (**c**). The length of LDECs was 76.5 ± 8.1 µm in the control group, 8.41 ± 0.53 µm in the 1% BAC and vehicle injected group, and 63.3 ± 4.4 µm in the 1% BAC and rebamipide injected group (**d**) (*p < 0.05, Bonferroni correction). All tissues were stained with hematoxylin and eosin. Black arrows indicate basement membrane and white arrows indicate neutophils. Scale bar = 50 µm.

We next investigated the effect of rebamipide on 1% BAC-damaged LDECs *in vivo* (Fig. 2). We found that the LDECs were disrupted and the neutrophils were accumulated under the connective tissue in the group injected with 1% BAC and the vehicle of rebamipide (Fig. 2b). However, those inflammation-associated tissue disorders were not observed in the group injected with 1% BAC and rebamipide (Fig. 2c). The thickness of the LDECs in the control group, the 1% BAC and the vehicle of rebamipide injected group, and the rebamipide injected group was 76.5 ± 8.1 μm, 8.41 ± 0.53 μm, and 63.3 ± 4.4 μm, respectively (*p* < 0.05) (Fig. 2d).

**Figure 3:**
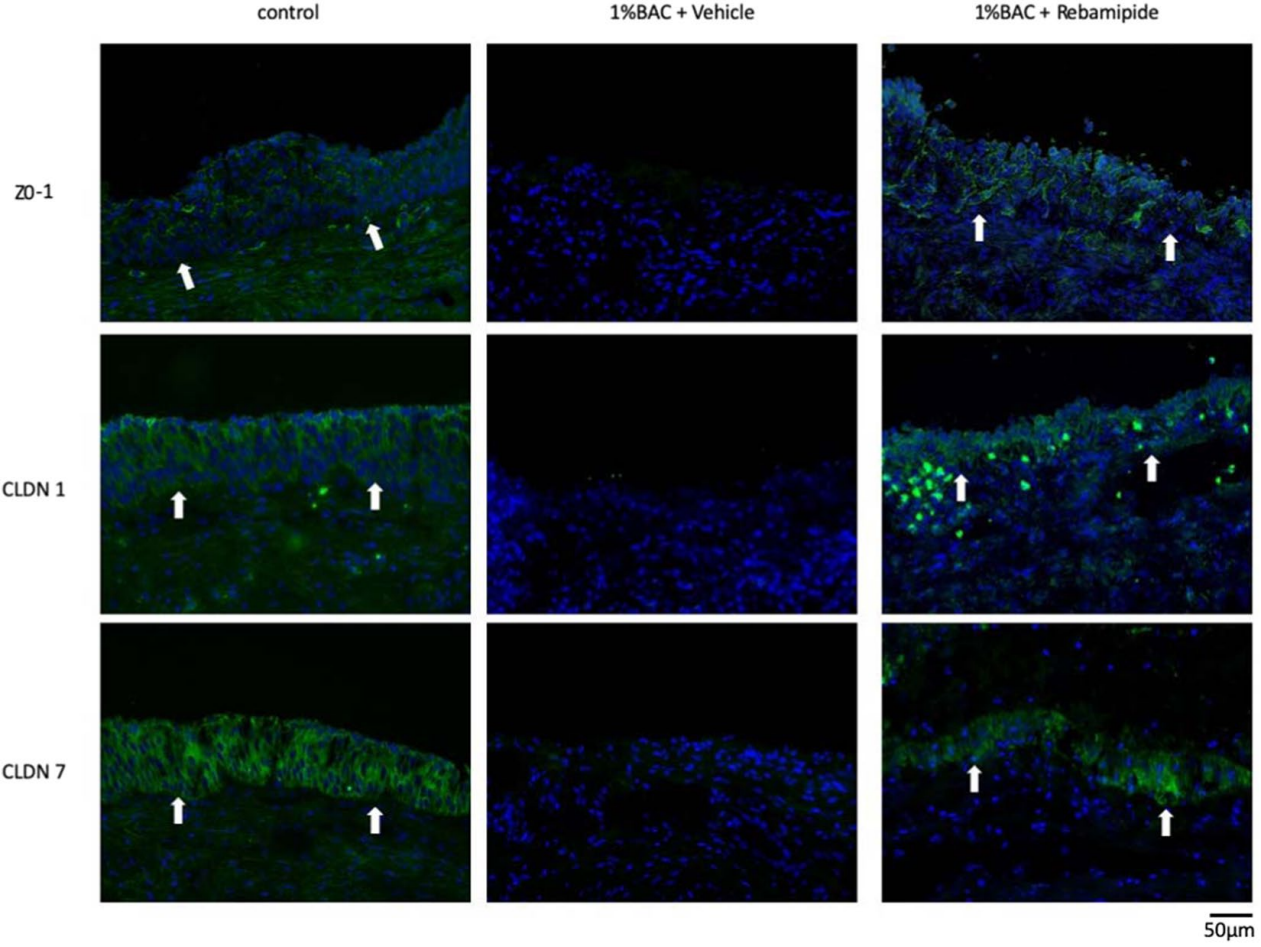
Tight junction protein expression of rabbit LDECs *in vivo* (n = 3 each). Tight junction proteins, including zonula occludens (ZO)-1, claudin (CLDN)1, and CLDN7 in rabbit LDECs were disrupted in the 1% BAC and the vehicle of rebamipide injected group (middle) and preserved in the 1% BAC and rebamipide injected group (right) as well as in the control group (left). White arrows indicate basement membrane. Scale bar = 50 µm.

Immunohistological examination findings confirmed the expression of tight junction proteins ZO-1, CLDN1, and CLDN7 between the epithelial cells in the control group and in the 1% BAC and rebamipide injected group, yet disruption in the 1% BAC and vehicle of rebamipide injected group (Fig. 3).

**Figure 4:**
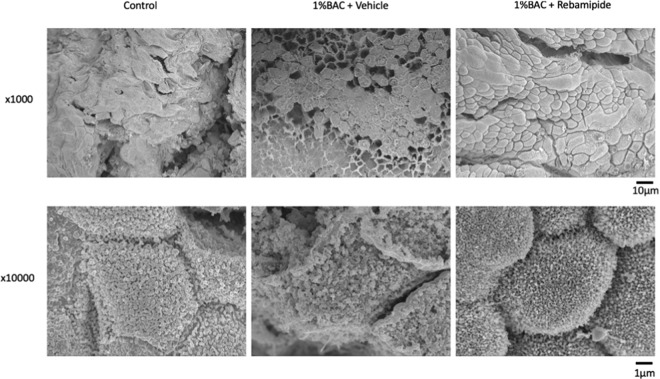
Analysis of the SEM images on the surface of the LDECs *in vivo* (n = 1 each). In scanning electron microscopy examination, LDECs were lined disrupted and microvilli of cells were shortened in the 1% BAC and the vehicle of rebamipide injected group to compare the control group (upper row × 1,000 and lower row × 10,000). The surface of LDECs was smooth and microvilli of cells were lined orderly in the 1% BAC and rebamipide injected group. Scale bars = 10 µm and 1 µm.

Analysis of the SEM images revealed healthy microvilli on the surface of the LDECs in the group injected with 1% BAC and rebamipide, similar to the findings observed in the control group. On the other hand, healthy microvilli were not observed accompanied with a disruption of the LDECs in the group injected with 1% BAC and the vehicle of rebamipide (Fig. 4).

## Discussion

In this study, we successfully cultivated LDECs *in vitro* and established a rabbit lacrimal-duct-damage model with BAC *in vivo*. Our findings revealed that rebamipide attenuates the IL-6-induced decrease of barrier function between rabbit LDECs *in vitro*, and that it has protective effects on rabbit LDECs in a BAC-induced lacrimal-duct-damage model that disrupts the tight junctions between the cells *in vivo*. These results suggest that rebamipide suppresses the inflammatory-induced barrier function damage and helps to maintain the LDEC structure, thus protecting against tissue disruption.

Although numerous clinical research and anatomy-related studies focusing on lacrimal ducts have previously been performed, there have been few published basic-research reports on the related cell biology. In this present study, we were able to successfully cultivate LDECs until confluence, and subculture the LDECs using the method previously described by Xie *et al*.^6^.

Inflammation is thought to be a pathology of NLDO, and it has been reported that several inflammatory cytokines, such as IL-2, IL-6, IL-10, vascular endothelial growth factor, and fibroblast growth factor 2, are increased in the tear fluid of patients with PANDO^2^, and that IL-6, specifically, is increased in the tear fluid of patients with CNLDO^3^. As the findings in several previous reports have suggested that IL-6 attenuates the barrier function between cells^7,8^, our results revealed that IL-6 induced barrier dysfunction of cultured LDECs. In this present study, our findings suggest that the anti-inflammatory effect of rebamipide can maintain intercellular barrier function by suppressing IL-6 inflammation. Based on these results, anti-inflammatory drugs might possibly be one key element in the treatment of lacrimal-duct disease.

Several studies on the anatomy of lacrimal ducts in various animals have been reported, and the findings in those studies have shown that rabbit lacrimal ducts are suitable for creating models to clarify disease pathology, as the anatomy of those ducts is similar to human lacrimal ducts^6,9^. Via the injection of BAC, we were able to successfully create a rabbit lacrimal duct damage model. Histological examination revealed obvious disruption of LDECs with the injection of 1% or more BAC. Thus, 1% was selected as the appropriate percentage of BAC to create the lacrimal duct damage model (Supplemental Fig. 2). BAC is frequently used as an ophthalmic preservative, and it is also known to cause ocular tissue damage^10,11^. In fact, the long-term use of anti-glaucoma eye drops, specifically, is known to cause inflammation and fibrosis-related changes in conjunctiva^12^, and is also known to be a risk factor for the development of NLDO^13^. Those changes may be related to the preservatives in the commercial preparations, and/or the medications themselves^14^. Hence, in this study, we chose to use a rabbit lacrimal duct damage model treated with BAC, and the infiltration of neutrophils was observed in addition to epithelial disorder in this model, thus indicating that BAC injection resulted in acute inflammation in the lacrimal duct. Moreover, our findings revealed that rebamipide also antagonizes the BAC-induced shedding of LDECs with anti-inflammatory actions.

It has been reported that rebamipide was first developed as a gastroprotective drug due to its ability to increase gastric mucus production^15,16^ and suppress gastric mucosal inflammation^17,18^. In Japan, it has also been used as an ophthalmic suspension for the treatment of dry eye. Previous studies have reported that rebamipide has an anti-inflammation effect on CECs^4^, corneal fibroblasts^19^, and conjunctival epithelial cells (CjECs)^20^. It has also been reported that rebamipide helps to maintain the microvilli of CECs and CjECs against N-acetylcysteine^21^ and the barrier function between CECs^4^.

Moreover, it has been reported that human LDECs are faced with microvilli^22^, the same as in rabbit LDECs. In this present study, we found that rebamipide also helps to maintain the microvilli of LDECs. In regard to lacrimal duct disease, it has been reported that rebamipide clinically improved postoperative results after tube intubation for PANDO^5^, however, the specific underlying mechanisms for that improvement are still unknown. In this present study, our findings showed that rebamipide improves the disruption of barrier function of LDECs, thus suggesting that its use post-surgery might improve surgical outcomes.

In summary, the findings in this study show that rebamipide has a protective effect against inflammatory cytokines that helps to recover the barrier function of LDECs *in vitro*, and that pre-administered rebamipide suppressed BAC-induced cell damage *in vivo*. Although rebamipide already has a long and well-recognized history of being applied for the treatment of corneal and gastro-intestinal diseases, maybe it can possibility now be repositioned as an effective drug for the protection of LDECs as well.

## Supplementary information

Supplementary Information 
