# Supplementary material for: Rebamipide promotes lacrimal duct epithelial cell survival via protecting barrier function
Source: Sci Rep. 2020 Feb 3;10:1641. doi: 10.1038/s41598-020-58314-x (PMC6997454; doi:10.1038/s41598-020-58314-x)

Rebamipide promotes lacrimal duct epithelial cell survival via protecting barrier function

Hiroshi Tanaka[1*], Tomomichi Nakayama[1], Michiko Tsukamoto[1], Akihide Watanabe[1], Takahiro Nakamura[2], Norihiko Yokoi[1], Chie Sotozono[1], Shigeru Kinoshita[2]

[1]Department of Ophthalmology, Kyoto Prefectural University of Medicine, Kyoto, Japan.

[2]Department of Frontier Medical Science and Technology for Ophthalmology, Kyoto Prefectural University of Medicine, Kyoto, Japan.

* **Address correspondence to:** Hiroshi Tanaka, MD, PhD, Department of Ophthalmology, Kyoto Prefectural University of Medicine, 465 Kajii-cho, Hirokoji-agaru, Kawaramachi-dori, Kamigyo-ku, Kyoto 602-0841, Japan.

Tel: +81-75-251-5578; Fax: +81-75-251-5663; e-mail: htanakan@koto.kpu-m.ac.jp

Supplemental Figure Legends

Supplemental Figure 1. In the immunohistological examination *in vitro* (n=2 each), ZO-1 and CLDN-7 were partially expressed in the control group (**a, g**) and the 1% BAC and rebamipide stimulated group (**c, i**), but not in the 1% BAC and vehicle of rebamipide stimulated group (**b, h**). CLDN-1 was expressed in the control group (**d**), but not in the 1% BAC and rebamipide stimulated group (**f**) and the 1% BAC and vehicle of rebamipide stimulated group (**i**). Scale bar = 50μm.

Supplemental Figure 2. Histological examination of LDECs in several concentrations of BAC *in vivo* (n=3 each). Normal LDECs are shown in control (0% BAC injection) (**a**), mild tissue damage, such as epithelial shortening and neutrophil infiltration were with 0.1% BAC injection (**b**) and disruption of LDECs are shown in 1% BAC injection (**c**) and 10% BAC injection (**d**). All tissues were stained with hematoxylin and eosin. The black arrows indicate basement membrane, and the white arrows indicate neutrophil accumulation. Scale bar = 50μm.

Supplemental Figure 1.

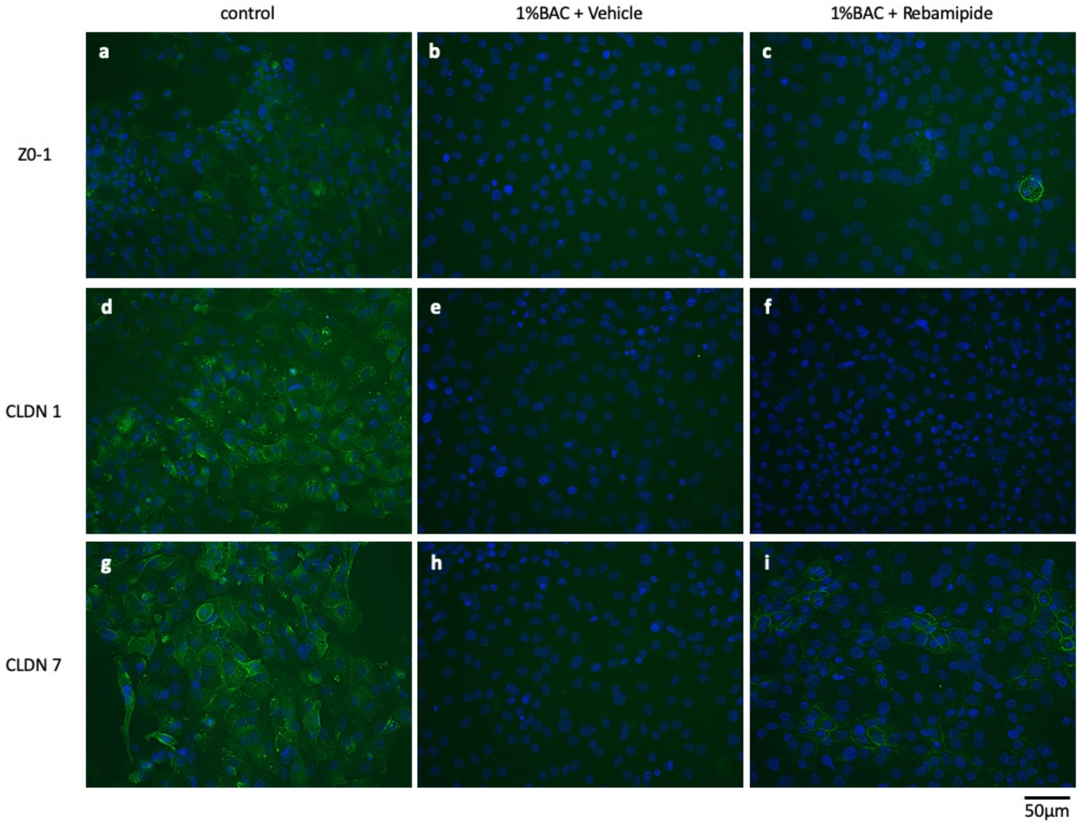

Supplemental Figure 2.

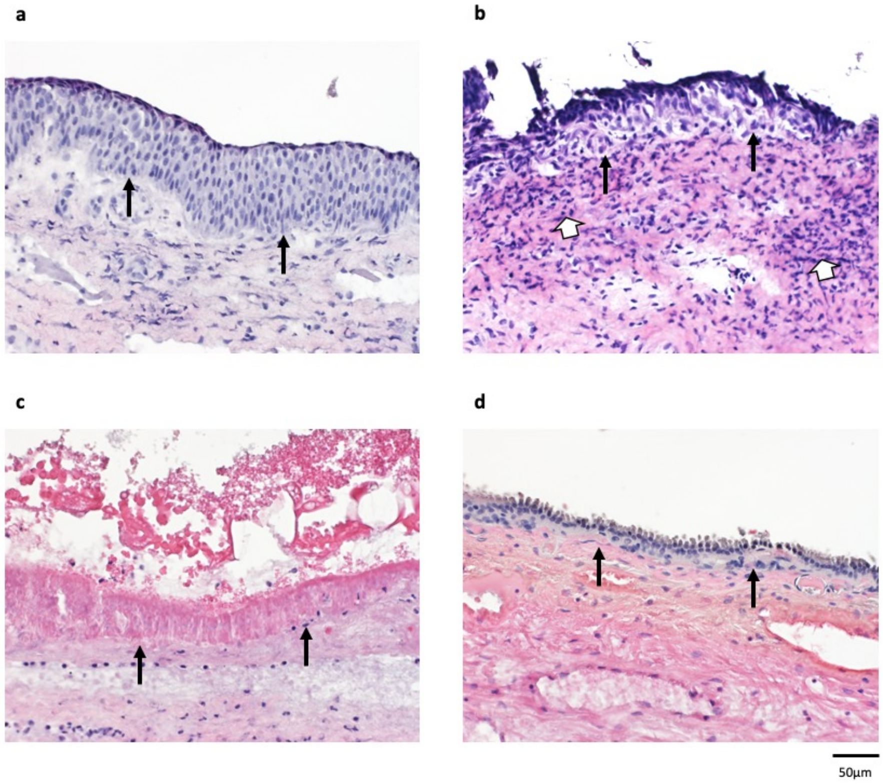

Supplement: Supplementary file 1 — Supplementary Information [file 41598_2020_58314_MOESM1_ESM.pdf]
